# Long-Term Clinical Outcomes Between Biodegradable and Durable Polymer Drug-Eluting Stents: A Nationwide Cohort Study

**DOI:** 10.3389/fcvm.2022.873114

**Published:** 2022-04-29

**Authors:** Seung-Jun Lee, Dong-Woo Choi, Yongsung Suh, Sung-Jin Hong, Chul-Min Ahn, Jung-Sun Kim, Byeong-Keuk Kim, Young-Guk Ko, Donghoon Choi, Eun-Cheol Park, Yangsoo Jang, Chung-Mo Nam, Myeong-Ki Hong

**Affiliations:** ^1^Severance Cardiovascular Hospital, Yonsei University College of Medicine, Seoul, South Korea; ^2^Department of Preventive Medicine, Yonsei University College of Medicine, Seoul, South Korea; ^3^Cancer Big Data Center, National Cancer Control Institute, National Cancer Center, Goyang, South Korea; ^4^Myongji Hospital, Hanyang University College of Medicine, Goyang, South Korea

**Keywords:** coronary artery disease, drug-eluting stent, percutaneous coronary intervention, treatment outcome, stents

## Abstract

**Background:**

Despite the theoretical benefits of biodegradable polymer drug-eluting stents (BP-DES), clinical benefits of BP-DES over durable polymer DES (DP-DES) have not been clearly demonstrated. Using data from a large-volume nationwide cohort, we compared long-term clinical outcomes between BP-DES- and DP-DES-treated patients.

**Methods:**

A retrospective cohort study that enrolled all patients who underwent percutaneous coronary intervention (PCI) with new-generation DES between 2010 and 2016 in Korea was conducted by using the National Health Insurance Service database. The outcomes of interest were all-cause death, cardiovascular death, and myocardial infarction (MI).

**Results:**

A total of 127,731 patients treated with new-generation DES with thin struts (<90 μm) were enrolled for this analysis. After stabilized inverse probability of treatment weighting, the incidence of all-cause death was significantly lower in patients treated with BP-DES (*n* = 19,521) at 5 years after PCI (11.3 vs. 13.0% in those treated with DP-DES [*n* = 108,067], hazard ratio [HR] 0.92, 95% confidence interval [CI], 0.88–0.96, *p* < 0.001), while showing no statistically significant difference at 2 years after PCI (5.7 vs. 6.0%, respectively, HR 0.95, 95% CI, 0.89–1.01, *p* = 0.238). Similarly, use of BP-DES was associated with a lower incidence of cardiovascular death (7.4 vs. 9.6% in those treated with DP-DES, HR 0.82, 95% CI, 0.77–0.87, *p* < 0.001), and MI (7.4 vs. 8.7%, respectively, HR 0.90, 95% CI, 0.86–0.94, *p* = 0.006) at 5 years after PCI. There was no statistically significant difference of cardiovascular death (4.6 vs. 4.9%, respectively, HR 0.93, 95% CI, 0.85–1.01, *p* = 0.120) and MI (5.0 vs. 5.1%, respectively, HR 0.98, 95% CI, 0.92–1.05, *p* = 0.461) at 2 years after PCI.

**Conclusions:**

Implantation of BP-DES was associated with a lower risk of all-cause death, cardiovascular death, and MI compared with DP-DES implantation. This difference was clearly apparent at 5 years after DES implantation.

**Clinical Trial Registration:**

ClinicalTrial.gov, NCT04715594.

## Introduction

Compared with first-generation drug-eluting stents (DES) that harbored the risk of stent thrombosis, new-generation DES with durable polymers (DP-DES) have successfully lowered the risk of stent thrombosis while maintaining the lower rate of in-stent restenosis, compared with bare-metal stents (1, 2). In addition, advances in stent manufacturing technology enabled the reduction of stent strut thickness while securing sufficient radial force, and the development of a biocompatible polymer that allows stable release of anti-proliferative drugs has significantly reduced the frequency of stent failure (3). Despite these advances in technology, very late stent thrombosis and neoatherosclerosis still contribute to late fatal clinical outcomes in some subjects who successfully underwent percutaneous coronary intervention (PCI) with new-generation DES (4). As a plausible explanation, persistence of polymer in the stent platform could continuously evoke chronic inflammation, delay endothelial healing, and accelerate neoatherosclerosis (4, 5). However, if the polymer disappears by gradual biodegradation within a certain period after PCI, additional polymer-related complications may not occur. Therefore, DESs with biodegradable polymers (BP-DES) have been developed and are currently being actively utilized in contemporary clinical practice (6). Despite the theoretical superiority of BP-DES over DP-DES, prior reports, including randomized trials that enabled the use of BP-DES in clinical practice, mostly failed to demonstrate the superiority of BP-DES compared with DP-DES in short-term periods (around 1-year follow-up) with a relatively insufficient number of study participants, considering the low rates of cardiac events after implantation of new-generation DES (7–10). To date, the clinical benefits of BP-DES over DP-DES are controversial (1, 7–11). In this regard, we sought to investigate the long-term clinical impact of BP-DES compared with DP-DES utilizing the large-volume nationwide cohort that covers the entire populations who received first- and new-generation DES implantation for coronary artery disease in Korea (CONNECT DES cohort registry).

## Materials and Methods

### Study Design and Data

This study was a retrospective analysis of the national health claims database established by the National Health Insurance Service (NHIS) of Korea. This database contains claimed medical cost, detailed information of prescribed drugs including the number of pills and drug dosage, and medical history presented as International Classification of Diseases, Tenth Revision (ICD-10) codes. Most of the Korean population (97.1%) are forced to subscribe to the NHIS, which is a sole insurer managed by the Korean government. Given that NHIS also covers information for the remaining population (2.9%) categorized as medical aid subjects, this cohort is considered to represent the entire Korean population (12). We were also provided with the death certificates with ICD-10 codes from the National Institute of Statistics of Korea. This study was approved by the Institutional Review Board of our institute. Informed consent was waived because personal information was masked after cohort generation according to strict confidentiality guidelines of the Korean Health Insurance Review and Assessment Service. This study is registered at ClinicalTrial.gov (NCT04715594).

### Study Population and Covariates

**Figure 1:**
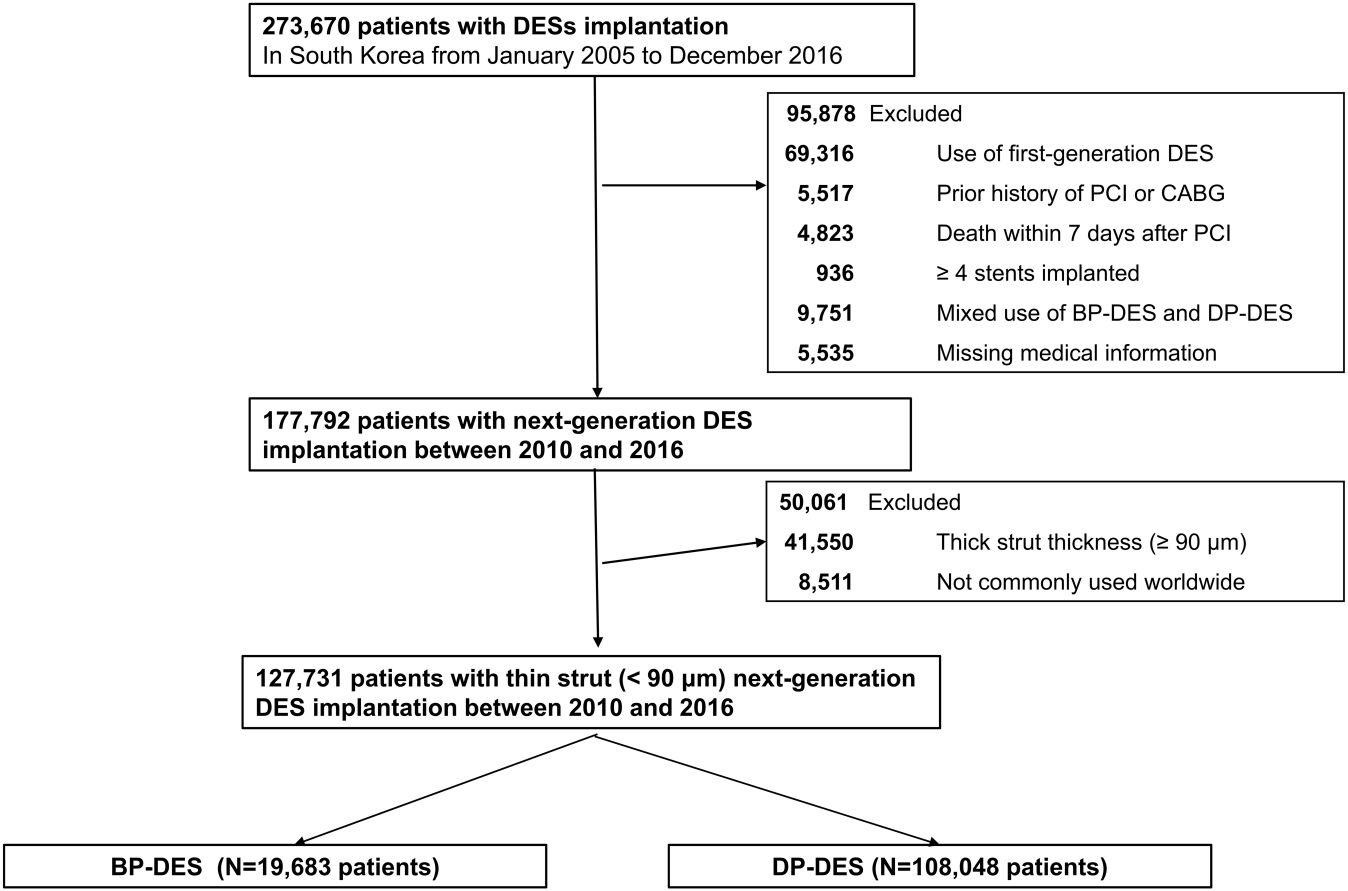
A flowchart of study population. DES, drug-eluting stent; BP-DES, biodegradable polymer drug-eluting stent; CABG, coronary artery bypass graft; DP-DES, durable polymer drug-eluting stent; PCI, percutaneous coronary intervention.

Among about 51.5 million inhabitants included in the Korean NHIS database, we recruited 273,670 patients (≥20 years old) who were treated with DES between January 2005 and December 2016 in Korea (CONNECT DES cohort registry). First-generation DES implantation was more frequently performed between 2005 and 2009. New-generation DESs were more frequently implanted between 2010 and 2016. According to the stringent policy of the NHIS database to protect personal information, type of polymer, generation of DES, strut thickness and type of eluted drugs were only provided without the DES product names. Figure 1 shows the study flow. DES implantation was performed in 273,670 patients between 2005 and 2016. Among 273,670 patients, 95,878 patients were excluded from this study: patients who were implanted with first-generation DES (*n* = 69,316); those who had a prior history of PCI or coronary artery bypass surgery (*n* = 5,517) because clinical events during follow-up cannot be discriminated whether those were caused by a prior PCI (coronary artery bypass surgery) or index PCI; those who died within 7 days after index PCI (*n* = 4,823) because these early death might be not due to different DESs but to clinical characteristic of the patients; those with four or more stents implanted (*n* = 936); those who were implanted with both types of DES (*n* = 9,751); and those with missing medical information (*n* = 5,535). Therefore, 177,792 patients who were treated with new-generation DES remained. Among these patients, those implanted with new-generation DES with thick struts (≥90 μm thickness) (*n* = 41,550) or struts not commonly used worldwide (*n* = 8,511) were further excluded. Consequently, the remaining 127,731 patients who were treated with new-generation DES with thin struts (BP-DES, 19,683 patients and DP-DES, 108,048 patients) were finally included in the analysis of this study (Figure 1). The list of included or excluded new-generation DES are presented in Supplementary Table 1.

### Study Procedures and Outcomes

We utilized the ICD-10 codes, fee-for-service, and prescribed drug codes that were claimed during the study period provided by NHIS database, and death-certificates provided by the National Statistical Office. The NHIS database was reviewed and evaluated for the appropriateness of medication prescriptions across the country, which enabled accurate monitoring of drug compliance and prescription status (13, 14). The clinical outcomes of interest were all-cause death, cardiovascular death, and myocardial infarction (MI). Cardiovascular death was ascertained from the National Statistical Office of Korea, which provided the death certificates with an accuracy of 92% for specific cause of death (12, 15). Cardiovascular death was identified by a death certificate with at least one cardiovascular-related diagnosis (acute MI, stroke, heart failure or sudden cardiac death) (16). MI was defined by the ICD-10 codes corresponding to acute MI (14), and satisfying one or more of the following conditions: (1) concurrent presence of claims for coronary angiography, (2) admission *via* emergency department, or (3) performance of cardiac biomarker testing more than 4 times. Additionally, we included baseline comorbidities and drug prescription status before PCI for the propensity score calculation, and inverse probability treatment of weighting (IPTW) was used to account for differences in baseline characteristics, medical history and confounding bias (13, 16). Details regarding covariates included in the propensity score calculation are described in Supplementary Table 2.

### Sensitivity Analysis

In order to assess the consistency of our analyses, we performed subgroup analyses for all-cause death, cardiovascular death, or MI stratified by age, sex, hypertension, diabetes mellitus, presentation as acute MI, chronic kidney disease with severe renal impairment, and prior cerebrovascular accidents.

### Statistical Analysis

Continuous variables are reported as mean and standard deviation, while dichotomous variables are presented as frequencies and their percentages. To minimize the effect of confounding bias, we calculated the inverse probability of treatment weights (IPTW) by the propensity score, which was calculated by logistic regression with covariates including age, sex, history of comorbidities and medications, and year of PCI (Supplementary Table 2). We also stabilized the weights by multiplying IPTW by the marginal probability of receiving treatment. The effect size difference between the two groups for all comorbidities and medications was calculated using the standardized mean difference (SMD) and Kernel density plots. SMD values above 0.2 were regarded as potential imbalance between the two groups. Cumulative incidence curves and the rate of the clinical outcomes of interest during follow-up were plotted using the Kaplan–Meier method. The adjusted hazard ratio (HR) for each clinical outcome of interest was calculated using a Cox proportional hazard regression model. Cause-specific hazard model was used to consider death as a competing risk when comparing the incidences of cardiovascular death or MI. The variables that were not balanced between the groups after IPTW-adjustment such as “year of PCI” and “duration of DAPT” were incorporated as covariates for multivariable regression analyses. A two-sided *p*-values of <0.05 were considered significant. Statistical analyses were conducted using SAS version 9.4 (SAS Institute, Cary, NC, USA) and R version 3.6 (The R Foundation, www.R-project.org).

## Results

**Figure 2:**
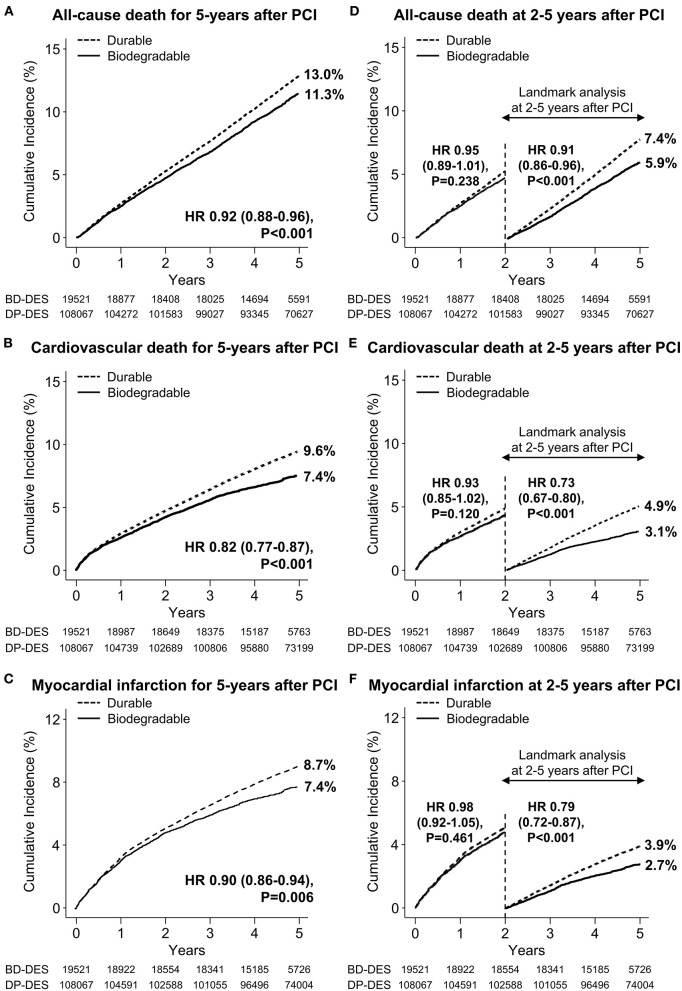
Time-to-event curves for the clinical outcome of interest and landmark analysis between 2 and 5 years after PCI. The cumulative incidence of **(A)** all-cause death, **(B)** cardiovascular death, and **(C)** myocardial infarction for 5 years after new-generation drug-eluting stent implantation. Landmark analyses between 2 and 5 years after index percutaneous coronary intervention for the cumulative incidence of **(D)** all-cause death, **(E)** cardiovascular death, and **(F)** myocardial infarction are presented. BP-DES, biodegradable polymer drug-eluting stent; DES, drug-eluting stent; DP-DES, durable polymer drug-eluting stent; HR, hazard ratio.

**Figure 3:**
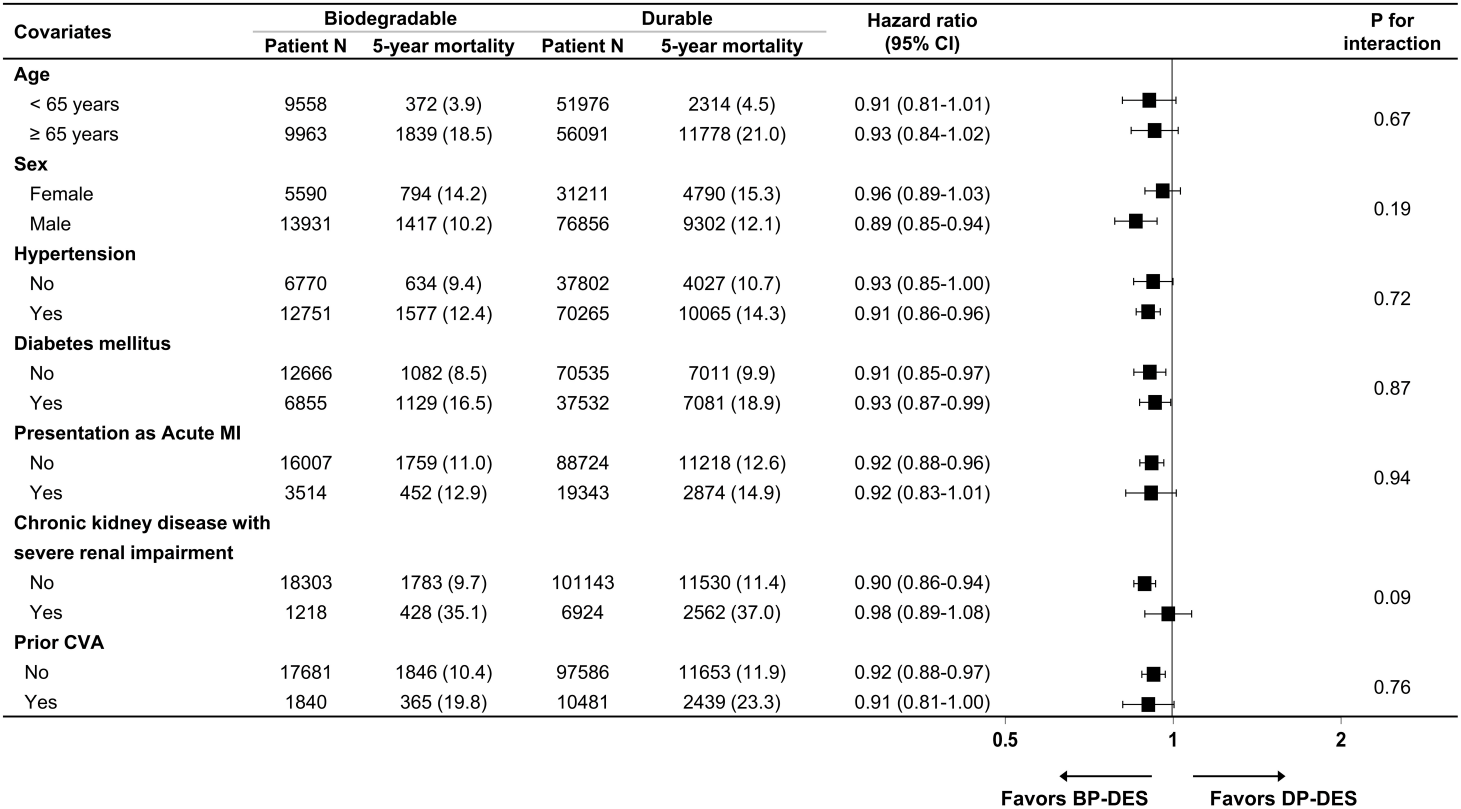
Subgroup analyses for all-cause death. Numbers and percentages show the number of patients at risk, those who died with any cause of death, and the all-cause mortality rate at 5 years after drug-eluting stent implantation. CI, confidence interval; CVA, cerebrovascular accidents; BP-DES, biodegradable polymer drug-eluting stent; DP-DES, durable polymer drug-eluting stent; MI, myocardial infarction.

**Figure 4:**
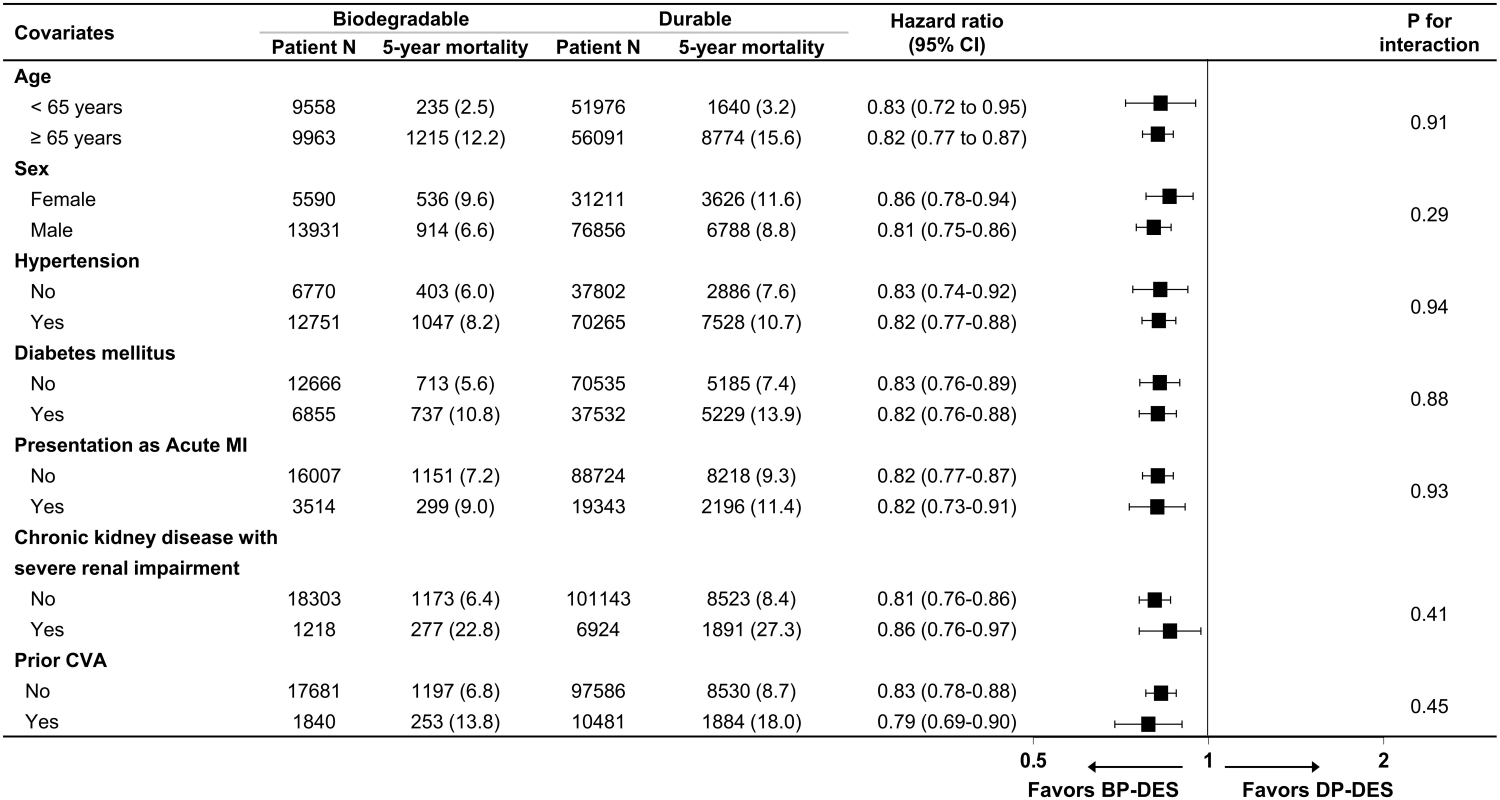
Subgroup analyses for cardiovascular death. Numbers and percentages show the number of patients at risk, those who died with cardiovascular disease, and the cardiovascular mortality rate at 5 years after drug-eluting stent implantation. CI, confidence interval; CVA, cerebrovascular accidents; BP-DES, biodegradable polymer drug-eluting stent; DP-DES, durable polymer drug-eluting stent; MI, myocardial infarction.

Baseline clinical characteristics and medical history of the whole cohort population before and after stabilized IPTW are presented in Table 1. After stabilized IPTW, there was no evidence of inequality in the baseline clinical characteristics and medications between the two groups (all SMD <0.1, Supplementary Figures 1, 2), except for the duration of dual antiplatelet therapy (DAPT) and year of index PCI. The incidence and relative hazards for the clinical outcomes of interest between the two groups after stabilized IPTW are presented in Table 2. At 5 years after index PCI, the incidence of all-cause death was significantly lower in patients treated with BP-DES (11.3 vs. 13.0% in those treated with DP-DES, HR 0.92, 95% CI, 0.88–0.96, *p* < 0.001; Figure 2A), while showing no statistically significant difference at 2 years after PCI (5.7 vs. 6.0%, respectively, HR 0.95, 95% CI, 0.89–1.01, *p* = 0.238). Statistical significance of reduced all-cause death was achieved in the BP-DES group at 3 years after index PCI (7.7 vs. 8.4% in DP-DES group, HR 0.93, 95% CI, 0.87–0.99, *p* = 0.015). Similarly, use of BP-DES was associated with a lower incidence of cardiovascular death (7.4 vs. 9.6% in those treated with DP-DES, HR 0.82, 95% CI, 0.77–0.87, *p* < 0.001; Figure 2B), and MI (7.4 vs. 8.7% in those treated with DP-DES, HR 0.90, 95% CI, 0.86–0.94, *p* = 0.006; Figure 2C) at 5 years after index PCI, while showing no statistically significant difference at 2 years after index PCI (4.6 vs. 4.9%, respectively, HR 0.93, 95% CI, 0.85–1.01, *p* = 0.120 for cardiovascular death; 5.0 vs. 5.1%, respectively, HR 0.98, 95% CI, 0.92–1.05, *p* = 0.461 for MI). The incidence and relative hazards for the outcomes of interest between the two groups before stabilized IPTW are presented in Supplementary Table 3. In a landmark analysis between 2 and 5 years after index PCI, the use of BP-DES was distinctly associated with reduced occurrence of all-cause death (5.9 vs. 7.4% in patients treated with DP-DES, HR 0.91, 95% CI, 0.86–0.96, *p* < 0.001; Figure 2D), cardiovascular death (3.1 vs. 4.9%, respectively, HR 0.73, 95% CI, 0.67–0.80, *p* < 0.001; Figure 2E) and MI (2.7 vs. 3.9%, respectively, HR 0.79, 95% CI, 0.72–0.87, *p* < 0.001; Figure 2F) (Supplementary Table 4). Multivariable regression analysis also revealed consistent favorable impact of BP-DES compared with DP-DES on all-cause or cardiovascular death at 5-years after PCI (Supplementary Tables 5, 6). A subgroup analysis showed that BP-DES had a consistent beneficial effect on the 5-year incidence of all-cause death (Figure 3), cardiovascular death (Figure 4), or MI (Supplementary Figure 3) across subgroups.

**Table 1:** Baseline characteristics and medications at discharge after index PCI.

	**Before stabilized IPTW (*N* = 127,731)**			**After stabilized IPTW (*N* = 127,588)**		
	**BP-DES** **(*N* = 19,683)**	**DP-DES** **(*N* = 108,048)**	**SMD**	**BP-DES** **(*N* = 19,521)**	**DP-DES** **(*N* = 108,067)**	**SMD**
Age, years	64.8 ± 11.7	64.7 ± 11.5	0.013	64.6 ± 11.6	64.7 ± 11.5	0.005
Women	5,405 (27.5)	31,494 (29.1)	0.037	5,590 (28.6)	31,211 (28.9)	0.005
**Comorbidity**						
Hypertension	12,301 (62.5)	70,798 (65.5)	0.063	12,750 (65.3)	70,265 (65.0)	0.006
Dyslipidemia	8,573 (43.6)	42,647 (39.5)	0.083	7,852 (40.2)	43,375 (40.1)	0.002
Diabetes mellitus	6,902 (35.1)	37,518 (34.7)	0.007	6,855 (35.1)	37,532 (34.7)	0.008
Chronic kidney disease with severe renal impairment^*^	1,334 (6.8)	6,861 (6.3)	0.017	1,218 (6.2)	6,924 (6.4)	0.007
Heart failure	2,680 (13.6)	14,960 (13.8)	0.007	2,666 (13.7)	14,925 (13.8)	0.004
Chronic liver disease	1,820 (9.2)	10,340 (9.6)	0.011	1,835 (9.4)	10,284 (9.5)	0.004
Chronic pulmonary disease	1,292 (6.6)	7,823 (7.2)	0.027	1,358 (7.0)	7,707 (7.1)	0.007
Peripheral arterial occlusive disease	731 (3.7)	4,028 (3.7)	0.001	734 (3.8)	4,020 (3.7)	0.002
Atrial fibrillation or flutter	734 (3.7)	3,802 (3.5)	0.011	708 (3.6)	3,835 (3.5)	0.004
Prior malignancy	1,052 (5.3)	4,895 (4.5)	0.038	917 (4.7)	5,028 (4.7)	0.002
Prior stroke or TIA	1,761 (8.9)	10,636 (9.8)	0.031	1,840 (9.4)	10,481 (9.7)	0.009
Prior ICH	114 (0.6)	550 (0.5)	0.010	92 (0.5)	557 (0.5)	0.006
Presentation as AMI	3,719 (18.9)	19,037 (17.6)	0.033	3,514 (18.0)	19,343 (17.9)	0.011
Thyroid disorder	530 (2.7)	2,923 (2.7)	0.001	551 (2.8)	2,924 (2.7)	0.007
Osteoporosis	1,296 (6.6)	7,574 (7.0)	0.017	1,364 (7.0)	7,492 (6.9)	0.002
**Medication prior to PCI**						
Anticoagulant	763 (3.9)	3,590 (3.3)	0.030	693 (3.5)	3,680 (3.4)	0.008
Anti-platelet agent	9,311 (47.3)	54,635 (50.6)	0.065	9,721 (49.8)	54,102 (50.1)	0.020
BP-lowering agents^†^	12,625 (64.1)	69,117 (64.0)	0.004	12,510 (64.1)	69,118 (64.0)	0.002
β-Blockers	13,559 (68.9)	78,945 (73.1)	0.092	14,148 (72.5)	78,217 (72.4)	0.002
RAAS blockade	12,414 (63.1)	72,293 (66.9)	0.081	12,619 (64.6)	71,577 (66.2)	0.013
Statin	18,681 (94.9)	102,711 (95.1)	0.007	18,584 (95.2)	102,664 (95.0)	0.007
**Duration of DAPT after PCI**						
<12 months	6,349 (32.1)	26,904 (24.9)	0.175	5,845 (30.5)	27,827 (25.7)	0.104
≥12 months	13,334 (67.9)	81, 144 (75.1)		13,676 (69.5)	80,240 (74.3)	
**Year of PCI**						
2010	145 (0.7)	14,861 (13.8)	1.178	212 (1.1)	14,702 (13.6)	1.190
2011	171 (0.9)	11,721 (10.8)		252 (1.3)	11,630 (10.8)	
2012	220 (1.1)	10,054 (9.3)		291 (1.5)	9,962 (9.2)	
2013	417 (2.1)	9,339 (8.6)		404 (2.1)	9,306 (8.6)	
2014	2,557 (13.0)	18,583 (17.2)		2,041 (10.5)	18,553 (17.2)	
2015	4,332 (22.0)	22,011 (20.4)		3,815 (19.5)	22,193 (20.5)	
2016	11,841 (60.2)	21,479 (19.9)		12,506 (64.1)	21,721 (20.1)	

*Values are the mean ± standard deviation or n (%). AMI, acute myocardial infarction; BP-DES, biodegradable polymer drug-eluting stent; BP, blood pressure; DP-DES, durable polymer drug-eluting stent; ICH, intracranial hemorrhage; IPTW, inverse probability of treatment weighting; PCI, percutaneous coronary intervention; RAAS, renin-angiotensin-aldosterone-system; SMD, standardized mean difference; TIA, transient ischemic attack; DAPT, dual antiplatelet therapy.*

* *Chronic kidney disease with advanced stage requiring intensive medical therapy and financial assistance from health insurance.*

† *Alpha receptor antagonists, calcium-channel blocker or diuretics*.

**Table 2:** Risk of clinical outcome between biodegradable and durable polymer DES after stabilized inverse probability of treatment weighting.

	**Follow-up time**	**BP-DES (*N* = 19,521)**	**DP-DES (*N* = 108,067)**	R0isk difference (95% CI)^*^	**Hazard ratio (95% CI)^†^**	***P*-value**
All-cause death	1 year	644 (3.3%)	3,795 (3.5%)	−0.2 (−0.5 to 0.1)	0.94 (0.86 to 1.02)	0.322
	2 year	1,113 (5.7%)	6,484 (6.0%)	−0.3 (−0.7 to 0.1)	0.95 (0.89 to 1.01)	0.238
	3 year	1,496 (7.7%)	9,040 (8.4%)	−0.7 (−1.1 to −0.3)	0.93 (0.87 to 0.99)	0.015
	4 year	1,989 (10.2%)	11,810 (10.9%)	−0.7 (−1.2 to −0.2)	0.92 (0.87 to 0.97)	<0.001
	5 year	2,211 (11.3%)	14,092 (13.0%)	−1.7 (−2.2 to −1.2)	0.92 (0.88 to 0.96)	<0.001
Cardiovascular death	1 year	547 (2.8%)	3,242 (3.0%)	−0.2 (−0.5 to 0.1)	0.93 (0.85 to 1.02)	0.137
	2 year	898 (4.6%)	5,295 (4.9%)	−0.3 (−0.6 to 0.0)	0.93 (0.85 to 1.01)	0.120
	3 year	1,146 (5.9%)	7,262 (6.7%)	−0.8 (−1.2 to −0.4)	0.87 (0.82 to 0.93)	<0.001
	4 year	1,357 (7.0%)	9,045 (8.4%)	−1.4 (−1.9 to −0.9)	0.83 (0.78 to 0.88)	<0.001
	5 year	1,450 (7.4%)	10,414 (9.6%)	−2.2 (−2.7 to −1.7)	0.82 (0.77 to 0.87)	<0.001
Myocardial infarction	1 year	599 (3.1%)	3,476 (3.2%)	−0.1 (−0.4 to 0.1)	0.96 (0.88 to 1.05)	0.783
	2 year	967 (5.0%)	5,479 (5.1%)	−0.1 (−0.4 to 0.2)	0.98 (0.92 to 1.05)	0.461
	3 year	1,179 (6.0%)	7,012 (6.5%)	−0.4 (−0.8 to 0.0)	0.94 (0.88 to 1.00)	0.394
	4 year	1,378 (7.1%)	8,405 (7.8%)	−0.7 (−1.1 to −0.3)	0.92 (0.87 to 0.97)	0.037
	5 year	1,447 (7.4%)	9,435 (8.7%)	−1.3 (−1.7 to −0.9)	0.90 (0.86 to 0.94)	0.006

*BP-DES, biodegradable polymer drug-eluting stents; DP-DES, durable polymer drug-eluting stent; CI, confidence interval. Number in parentheses represent the percentage.*

* *Risk difference (95% CI) were calculated by Poisson regression with identity link.*

† *Hazard ratios (95% CI) were calculated by Cox proportional hazard model*.

## Discussion

To the best of our knowledge, the results of our analyses were derived from a nationwide cohort with the largest study population ever published. Of note, a major strength of our study was inclusion of all patients treated with thin-strut DESs that are commonly used world-wide in daily clinical practice. Therefore, very-high-risk patients who were usually excluded in prior randomized studies were entirely included in this study. In addition, there was completeness in monitoring for demographic characteristics, comorbidities, medication history, occurrence of clinical events requiring hospitalization, and prescription status of essential cardiac medications such as anti-platelet agents during 5-year follow-up after index PCI. Through analysis of the death certificates provided by the National Statistical Office, we could discriminate cardiovascular death from all-cause death. Therefore, we could investigate the impact of remnant polymer in new-generation DES on cardiovascular death. The principal findings of our study are as follows: (1) In the early term period, the rates for the clinical outcomes of interest were not significantly different between BP- and DP-DES-treated patients; however, (2) the cumulative incidence of clinical events was gradually different between the two groups as time passed after index PCI. Favorable results for the clinical outcomes of interest were distinctly apparent at 5 years after index PCI in BP-DES-treated patients.

It has been suggested that anti-proliferative drugs *per se* and their polymer carriers in the coronary arterial bed continuously evoke chronic vascular inflammation (4, 5) that leads to delayed vascular healing and incomplete endothelial strut coverage and potentially contributes to clinical presentation of very late stent thrombosis and MI (17). Autopsy studies elucidated a more robust extra-cellular matrix deposition in the re-stenotic lesions of implanted DP-DES compared with bare-metal stents (18), which are considered to play a crucial role in the development of neoatherosclerosis and consequent stent failure (4).

Prior randomized clinical trials and meta-analyses comparing BP- and DP-DES have demonstrated confounding results (6–9, 11). The BIOFLOW V randomized trial showed a significant clinical benefit of biodegradable polymer sirolimus-eluting stents (*n* = 884 patients) over the durable polymer everolimus-eluting stents (*n* = 450 patients) by reducing 1-year incidence rate of target-lesion failure (11). However, the Bio-RESORT randomized trial showed no statistically significant difference of primary endpoint at 12 months between biodegradable polymer everolimus-eluting (*n* = 1,172 patients) and sirolimus-eluting stents (*n* = 1,173 patients) vs. durable polymer zotarolimus-eluting stents (*n* = 1,169) (9). A meta-analysis that included 19,886 patients derived from 16 randomized clinical trials found no statistically distinct benefit of BP-DES over DP-DES during a mean follow-up duration of 26 months (6). It might be inappropriate to prove the clinical benefits of BP-DES over DP-DES within 12 months after index PCI. Because DAPT for about 12 months was usually recommended in most studies, the complications or clinical presentations caused by persistent polymer in DP-DES might be masked by DAPT.

Neoatherosclerosis is one of the main mechanisms of very late stent thrombosis after DES implantation (19). Persistence of polymer of DP-DES in the coronary vascular bed may also provoke chronic inflammation characterized by infiltration of inflammatory cells such as macrophage or lymphocytes, playing a central role in the progression of neoatherosclerosis (4). And, the development of neoatherosclerosis is a time-dependent phenomenon; the frequency of neoatherosclerosis increases with the stent age. Our prior intracoronary optical coherence tomography study showed that neoatherosclerosis was observed in 45.7% of patients between 3 and 5 years, 61.5% between 5 and 7 years and 73.9% more than 7 years after DES implantation (20). Therefore, neoatherosclerosis observed at the very late period was significantly associated with very late stent thrombosis. Furthermore, since the polymer degradation are generally known to take about 6–24 months (21, 22), it could be difficult to detect a statistical difference in clinical outcomes according to the polymer properties in early period. These prior findings may provide a plausible explanation for the beneficial effects of BP-DES that appear after a certain period of time post-new generation DES implantation. Indeed, in a prior large nationwide cohort that compared the effect of BP- and DP-DES in real-world practice (total 57,487 patients; BP-DES-treated patients, 10,032 and DP-DES-treated patients, 47,455), there was no significant difference in the occurrence of all-cause mortality or myocardial infarction for 2 years after index PCI (10). Similarly, our analysis also revealed no statistically significant difference in the occurrence of the outcomes of interest at 2 years after new-generation DES implantation; however, significant differences were gradually observed at 3 years post-DES implantation, favoring BP-DES. Based on our prior optical coherence tomography analysis and this newly acquired nationwide claims data analysis, we may postulate that the adverse effects of permanent remnant polymer in the coronary artery start to appear at about 3 years post-PCI and become distinct at about 5 years post-PCI. In addition, the increase in risk of cardiovascular events due to permanent remnant polymer is statistically clear, but the degree is somewhat modest, so it may be necessary to analyze a very large number of patients in order to identify statistically clear differences. The BIOSCIENCE randomized trial reported that 5-year risk of target lesion failure was similar between biodegradable polymer sirolimus-eluting stent (*n* = 1,063 patients) vs. durable polymer everolimus-eluting stent (*n* = 1,056 patients) (23). The ISAR-TEST 4 randomized trial recently reported that the 10-year incidence of major adverse cardiac events was not statistically different between biodegradable polymer sirolimus-eluting stents (*n* = 1,299 patients) vs. durable polymer everolimus-eluting stents (*n* = 652 patients) (24). These studies did not show a clinical benefit of BP-DES over DP-DES at 5- or 10-year follow-up (23, 24). The main reason for failure to demonstrate the theoretical benefits of BP-DES over DP-DES in these clinical studies might be inappropriately small number of study populations. With the number of all-cause deaths during 5-year follow-up and a total sample size of 127,731, our study had about 82.1% power to detect an HR of 0.80 in the comparison of the BP-DES group with the DP-DES group at a two-tailed alpha level of 5.0%. Further, because in contrast to usual design of randomized controlled trials, we did not exclude the very high-risk patients, the incorporation of very high-risk patients could have attributed to demonstrating a clinical benefit of BP-DES that become distinct over time.

## Limitations

This study has several limitations. First, the findings from this observational study cannot be applied to establish causal relationships and persistent residual confounding factors should be considered in the interpretation of our results, although we tried to minimize the bias through IPTW. Furthermore, as the NHIS database does not contain laboratory parameters or clinical information except for the diagnostic codes, unmeasured variables could have affected the results of our analyses. Second, because this database does not include angiographic or procedural information, including the extent and complexity of coronary artery disease, the impact of high-risk procedural characteristics was not fully considered in the interpretation of our analyses. Furthermore, since the result of electrocardiography was not available in this database, the impact of clinical presentation such as ST-elevation MI or non-ST elevation MI was not considered in statistical analyses. Third, to protect patients' personal information and avoid unnecessary conflict with the device manufacturer, the NHIS database provides information of DESs after sufficient encryption work, which includes DES generation, polymer degradability, and strut thickness. Finally, the temporal difference between the groups still remained even after IPTW-adjustment. Thus, care should be taken in interpretation of our result, although we adjusted the temporal difference in all regression analyses. Therefore, further analyses such as impact of the eluted drug or stent material was not performed, which should be carried out through following research.

## Conclusions

In this nationwide cohort of all patients treated with new-generation DES in Korea, BP-DES implantation was associated with a lower risk of all-cause death, cardiovascular death or MI. This difference appeared around 3 years after DES implantation and became more distinct with time.

## Data Availability Statement

The data analyzed in this study is subject to the following licenses/restrictions: the datasets generated for the analyses are not publicly available because of strict government restrictions. Requests to access these datasets should be directed to M-KH, mkhong61@yuhs.ac.

## Ethics Statement

The studies involving human participants were reviewed and approved by Severance Hospital Institutional Review Board. The Ethics Committee waived the requirement of written informed consent for participation.

## Author Contributions

S-JL, D-WC, C-MN, and M-KH contributed to the conception and design and verified the data and conducted all analyses. S-JL and M-KH wrote the study protocol. D-WC and C-MN performed the programming to extract the data from the NHIS database. M-KH had full access to all the data in the study and take responsibility for the integrity of the data and the accuracy of the data analysis. YS, S-JH, C-MA, J-SK, B-KK, Y-GK, DC, E-CP, and YJ provided a critical review of manuscript. All authors read and approved on the final publication.

## Funding

This work was supported by the Cardiovascular Research Center, Seoul, South Korea.

## Conflict of Interest

The authors declare that the research was conducted in the absence of any commercial or financial relationships that could be construed as a potential conflict of interest.

## Publisher's Note

All claims expressed in this article are solely those of the authors and do not necessarily represent those of their affiliated organizations, or those of the publisher, the editors and the reviewers. Any product that may be evaluated in this article, or claim that may be made by its manufacturer, is not guaranteed or endorsed by the publisher.

## Abbreviations

CI: confidence interval
DES: drug-eluting stent
BP-DES: DES with biodegradable polymers
DP-DES: DES with durable polymers
HR: hazard ratio
IPTW: inversed probability of treatment weighting
MI: myocardial infarction
HIS: National Health Insurance Service
PCI: percutaneous coronary intervention
SMD: standardized mean difference.
