# Supplementary material for: Long-Term Clinical Outcomes Between Biodegradable and Durable Polymer Drug-Eluting Stents: A Nationwide Cohort Study
Source: Front Cardiovasc Med. 2022 Apr 29;9:873114. doi: 10.3389/fcvm.2022.873114 (PMC9098972; doi:10.3389/fcvm.2022.873114)
Supplement: Supplementary file 1 [file Data_Sheet_1.docx]

Supplementary Material

**Long-term clinical outcomes between biodegradable versus durable polymer drug-eluting stents: a nationwide cohort study**

Seung-Jun Lee, Dong-Woo Choi, Yongsung Suh, Sung-Jin Hong, Chul-Min Ahn, Jung-Sun Kim, Byeong-Keuk Kim, Young-Guk Ko, Donghoon Choi, Eun-Cheol Park, Yangsoo Jang, Chung-Mo Nam, Myeong-Ki Hong

| **Contents** | **Page** |
| --- | --- |
| **Table S1.** List of new-generation DES included or excluded from the study | 2 |
| **Table S2.** Covariates include in stabilized inverse probability of treatment weighting | 3 |
| **Table S3.** Risk of clinical outcomes between biodegradable and durable polymer DES before stabilized inverse probability of treatment weighting | 4 |
| **Table S4.** Risk of clinical outcomes between biodegradable and durable polymer DES by landmark analysis between 2 and 5 years after percutaneous coronary intervention. | 5 |
| **Table S5.** Multivariable predictors of all-cause death | 6 |
| **Table S6.** Multivariable predictors of cardiovascular death | 7 |
|  |  |
| **Figure S1.** Standardized difference before and after stabilized inverse probability of treatment weighting | 8 |
| **Figure S2.** Distribution of stabilized inverse probability of treatment weightings | 9 |
| **Figure S3.** Subgroup analyses for myocardial infarction | 10 |

**Table S1. List of new-generation DES included or excluded from the study**

| **Included** | **Excluded** |
| --- | --- |
| **Biodegradable polymer DES** | |
| Ultimaster | Nobori |
| Synergy | Biomatrix |
| Orsiro | Biomime |
|  | DESyne |
|  | Genoss |
| **Durable polymer DES** | |
| Xience Prime | Endeavor Sprint |
| Xience Xpedition | Endeavor ABT-578 eluting stent |
| Resolute Onyx |  |
| Resolute Integrity |  |
| Promus Element |  |
| Promus Element Plus |  |
| Promus Premier |  |

DES, drug-eluting stents.

**Table S2. Covariates include in stabilized inverse probability of treatment weighting**

|  | **Variable list** |
| --- | --- |
| **Comorbidities** | Age, gender, diabetes, hypertension, dyslipidemia, history of heart failure, presentation as acute myocardial infarction, prior history of stroke or cerebrovascular accident, prior history of intracranial hemorrhage, atrial fibrillation or flutter, prior history of malignancy, hyperthyroidism, hypothyroidism, osteoporosis, chronic liver disease, chronic pulmonary disease, chronic kidney disease with severe renal impairment |
| **Medications** | Warfarin sodium, Edoxaban, Rivaroxaban, Apixaban, Dabigatran, Aspirin, Clopidogrel, Prasugrel, Ticagrelor, Atorvastatin, Rosuvastatin, Simvastatin, Pravastatin, Fluvastatin, Pitavastatin, Lovastatin, Tripamol, Hydrochlorothiazide, Chlorthalidon, Indapamide, Metorazone, Furosemide, Torasemide, Amlodipine, Barnidipine, Cilnidipine, Felodipine, Lacidipine, Lercanidipine, Manidipine, Nicardipine, Nifedipine, Nisoldipine, Nitrendipine, Diltiazem, Verapamil, Bunazosin, Doxazosin, Prazosin, Terazosin, Atenolol, Betaxolol, Bevantolol, Bisoprolol, Celiprolol, Metoprolol, Nadolol, Propranolol, Carteolol, Arotinolol, Carvedilol, Labetalol, Sulfonylurea, Metformin, Alpha-glucosidase inhibitors, Thiazolidinedione, DPP-Ⅳ inhibitors, SGLT-2 inhibitors, insulin, Spironolactone, Alacepril, Benazepril, Captopril, Cilazapril, Delapril, Enalapril, Fosinopril, Imidapril, Lisinopril, Moexipril, Perindopril, Quinapril, Ramipril, Temocapril, Candesartan, Eprosartan, Irbesartan, Losartan, Telmisartan, Valsartan, Fimasartan, Azilsartan |

**Table S3** . **Risk of clinical outcomes between biodegradable and durable polymer DES before stabilized inverse probability of treatment weighting**

|  | **Follow-up time** | **BP-DES**  **(N=19,683)** | **DP-DES**  **(N=108,048)** | **Risk difference**  **(95% CI)**^*^ | **Hazard ratio**  **(95% CI)**^†^ | **P value** |
| --- | --- | --- | --- | --- | --- | --- |
| **All-cause death** | 1 year | 669 (3.4%) | 3,800 (3.5%) | -0.1 (-0.4 to 0.2) | 0.97 (0.89-1.05) | 0.415 |
|  | 2 year | 1,147 (5.8%) | 6,506 (6.0%) | -0.2 (-0.6 to 0.2) | 0.97 (0.91-1.03) | 0.306 |
|  | 3 year | 1,556 (7.9%) | 9,066 (8.4%) | -0.5 (-0.9 to 0.0) | 0.96 (0.91-1.00) | 0.417 |
|  | 4 year | 2,043 (10.4%) | 11,756 (10.9%) | -0.5 (-1.0 to 0.0) | 0.95 (0.91-0.99) | 0.029 |
|  | 5 year | 2,290 (11.6%) | 14,142 (13.1%) | -1.4 (-2.0 to -0.9) | 0.87 (0.83-0.91) | <0.001 |
| **Cardiovascular death** | 1 year | 568 (2.9%) | 3,333 (3.1%) | -0.2 (-0.5 to 0.1) | 0.94 (0.86-1.02) | 0.142 |
|  | 2 year | 928 (4.7%) | 5,401 (5.0%) | -0.3 (-0.6 to 0.0) | 0.94 (0.88-1.01) | 0.100 |
|  | 3 year | 1,220 (6.2%) | 7,294 (6.8%) | -0.6 (-1.0 to -0.2) | 0.92 (0.86-0.97) | 0.006 |
|  | 4 year | 1,449 (7.4%) | 9,085 (8.4%) | -1.0 (-1.4 to -0.6) | 0.88 (0.83-0.92) | <0.001 |
|  | 5 year | 1,549 (7.9%) | 10,463 (9.7%) | -1.8 (-2.3 to -1.3) | 0.85 (0.81-0.90) | <0.001 |
| **Myocardial infarction** | 1 year | 607 (3.1%) | 3,468 (3.2%) | -0.1 (-0.4 to 0.2) | 0.96 (0.88-1.05) | 0.363 |
|  | 2 year | 972 (4.9%) | 5,476 (5.1%) | -0.2 (-0.5 to 0.1) | 0.97 (0.91-1.04) | 0.456 |
|  | 3 year | 1,209 (6.1%) | 7,005 (6.5%) | -0.4 (-0.8 to 0.0) | 0.95 (0.89-1.01) | 0.083 |
|  | 4 year | 1,410 (7.2%) | 8,397 (7.8%) | -0.6 (-1.0 to -0.2) | 0.92 (0.87-0.98) | 0.005 |
|  | 5 year | 1,489 (7.6%) | 9,427 (8.7%) | -1.2 (-1.7 to -0.7) | 0.90 (0.85-0.95) | <0.001 |

Abbreviations: BP-DES, biodegradable polymer drug-eluting stents; DP-DES, durable polymer drug-eluting stent; CI, confidence interval. Number in parentheses represent the percentage. ^*^ Risk difference (95% CI) were calculated by Poisson regression with identity link. ^†^ Hazard ratios (95% CI) were calculated by Cox proportional hazard model.

**Table S4. Risk of clinical outcomes between biodegradable and durable polymer DES by landmark analysis between 2 and 5 years after percutaneous coronary intervention.**

|  | **Follow-up time** | **BP-DES**  **(N=183,35)** | **DP-DES**  **(N=100,441)** | **Risk difference**  **(95% CI)**^*^ | **Hazard ratio**  **(95% CI)**^†^ | **P value** |
| --- | --- | --- | --- | --- | --- | --- |
| **All-cause death** | 3 year | 402 (2.2%) | 2,517 (2.5%) | -0.3 (-0.5 to -0.1) | 0.87 (0.79 to 0.97) | 0.013 |
|  | 4 year | 863 (4.7%) | 5,156 (5.1%) | -0.4 (-0.7 to -0.1) | 0.92 (0.86 to 0.98) | 0.018 |
|  | 5 year | 1,081 (5.9%) | 7,480 (7.4%) | -1.6 (-2.0 to -1.2) | 0.91 (0.86 to 0.96) | <0.001 |
| **Cardiovascular death** | 3 year | 285 (1.6%) | 1,857 (1.9%) | -0.3 (-0.5 to -0.1) | 0.84 (0.74 to 0.95) | 0.006 |
|  | 4 year | 474 (2.6%) | 3,598 (3.6%) | -1.0 (-1.3 to -0.7) | 0.74 (0.67 to 0.82) | <0.001 |
|  | 5 year | 564 (3.1%) | 4,926 (4.9%) | -1.8 (-2.1 to -1.5) | 0.73 (0.67 to 0.80) | <0.001 |
| **Myocardial infarction** | 3 year | 213 (1.2%) | 1,533 (1.5%) | -0.4 (-0.6 to -0.2) | 0.77 (0.67 to 0.90) | 0.001 |
|  | 4 year | 412 (2.3%) | 2,926 (2.9%) | -0.6 (-0.8 to -0.4) | 0.79 (0.71 to 0.88) | <0.001 |
|  | 5 year | 488 (2.7%) | 3,957 (3.9%) | -1.2 (-1.5 to -0.9) | 0.79 (0.72 to 0.87) | <0.001 |

Abbreviations: BP-DES, biodegradable polymer drug-eluting stents; DP-DES, durable polymer drug-eluting stent; CI, confidence interval. Number in parentheses represent the percentage. ^*^ Risk difference (95% CI) were calculated by Poisson regression with identity link. ^†^ Hazard ratios (95% CI) were calculated by Cox proportional hazard model.

**Table S5. Multivariable predictors of all-cause death**

| **Variables** | **Univariate** | | **Multivariate** | |
| --- | --- | --- | --- | --- |
|  | **HR (95% CI)** | **P value** | **HR (95% CI)** | **P value** |
| BP-DES (vs. DP-DES) | 0.95 (0.91-0.99) | 0.013 | 0.90 (0.86-0.94) | <0.001 |
| Age ≥65 | 5.14 (4.93-5.35) | <0.001 | 4.37 (4.19-4.56) | <0.001 |
| Female | 1.30 (1.26-1.34) | <0.001 | 0.80 (0.78-0.83) | <0.001 |
| Hypertension | 1.37 (1.33-1.42) | <0.001 | 1.02 (0.98-1.05) | 0.368 |
| Diabetes mellitus | 2.00 (1.94-2.06) | <0.001 | 1.86 (1.80-1.92) | <0.001 |
| Chronic kidney disease with severe renal impairment | 2.25 (2.16-2.36) | <0.001 | 1.59 (1.52-1.66) | <0.001 |
| Heart failure | 2.22 (2.14-2.30) | <0.001 | 1.68 (1.62-1.74) | <0.001 |
| Peripheral arterial occlusive disease | 1.70 (1.59-1.81) | <0.001 | 1.33 (1.24-1.42) | <0.001 |
| Atrial fibrillation or flutter | 2.22 (2.09-2.36) | <0.001 | 1.19 (1.12-1.27) | <0.001 |
| Prior stroke or TIA | 2.09 (2.00-2.17) | <0.001 | 1.46 (1.40-1.52) | <0.001 |
| Presentation as AMI | 1.21 (1.17-1.26) | <0.001 | 1.45 (1.40-1.51) | <0.001 |
| DAPT duration (months) | 0.95 (0.94-0.96) | <0.001 | 0.95 (0.94-0.96) | <0.001 |
| Year of PCI | 0.99 (0.98-0.99) | 0.001 | 0.96 (0.95-0.97) | <0.001 |

Abbreviations: HR, hazard ratio; CI, confidence interval; BP-DES, biodegradable polymer drug-eluting stent; DP-DES, durable polymer drug-eluting stent; TIA, transient ischemic attack; AMI, acute myocardial infarction; DAPT, dual antiplatelet therapy; PCI, percutaneous coronary intervention.

**Table S6. Multivariable predictors of cardiovascular death**

| **Variables** | **Univariate** | | | **Multivariate** | | |
| --- | --- | --- | --- | --- | --- | --- |
|  | **HR (95% CI)** | **P value** | **HR (95% CI)** | | **P value** |  |
| BP-DES (vs. DP-DES) | 0.85 (0.81-0.90) | <0.001 | 0.87 (0.82-0.92) | | <0.001 |  |
| Age ≥65 years | 5.25 (5.00-5.52) | <0.001 | 4.26 (4.05-4.48) | | <0.001 |  |
| Female | 1.34 (1.29-1.39) | <0.001 | 0.83 (0.80-0.86) | | <0.001 |  |
| Hypertension | 1.43 (1.37-1.49) | <0.001 | 1.04 (1.00-1.09) | | 0.047 |  |
| Diabetes mellitus | 1.97 (1.90-2.04) | <0.001 | 1.78 (1.72-1.85) | | <0.001 |  |
| Chronic kidney disease with severe renal impairment | 2.27 (2.15-2.39) | <0.001 | 1.56 (1.48-1.65) | | <0.001 |  |
| Heart failure | 2.39 (2.29-2.49) | <0.001 | 1.77 (1.69-1.85) | | <0.001 |  |
| Peripheral arterial occlusive disease | 1.67 (1.54-1.80) | <0.001 | 1.29 (1.20-1.39) | | <0.001 |  |
| Atrial fibrillation or flutter | 2.44 (2.28-2.61) | <0.001 | 1.31 (1.23-1.41) | | <0.001 |  |
| Prior stroke or TIA | 2.17 (2.07-2.27) | <0.001 | 1.50 (1.43-1.57) | | <0.001 |  |
| Presentation as AMI | 1.26 (1.21-1.32) | <0.001 | 1.52 (1.45-1.59) | | <0.001 |  |
| DAPT duration (months) | 0.95 (0.94-0.96) | <0.001 | 0.95 (0.94-0.96) | | <0.001 |  |
| Year of PCI | 0.98 (0.97-0.99) | <0.001 | 0.93 (0.92-0.94) | | <0.001 |  |

Abbreviations: HR, hazard ratio; CI, confidence interval; AMI, acute myocardial infarction; BP-DES, biodegradable polymer drug-eluting stent; DP-DES, durable polymer drug-eluting stent; TIA, transient ischemic attack; DAPT, dual antiplatelet therapy; PCI, percutaneous coronary intervention.

**Figure S1.** Standardized difference before and after stabilized inverse probability of treatment weighting. The standardized mean difference for each covariate before (green) and after (red) stabilized inverse probability of treatment weighting are presented. Standardized mean difference lesser than 0.10 was considered to be balanced.

**
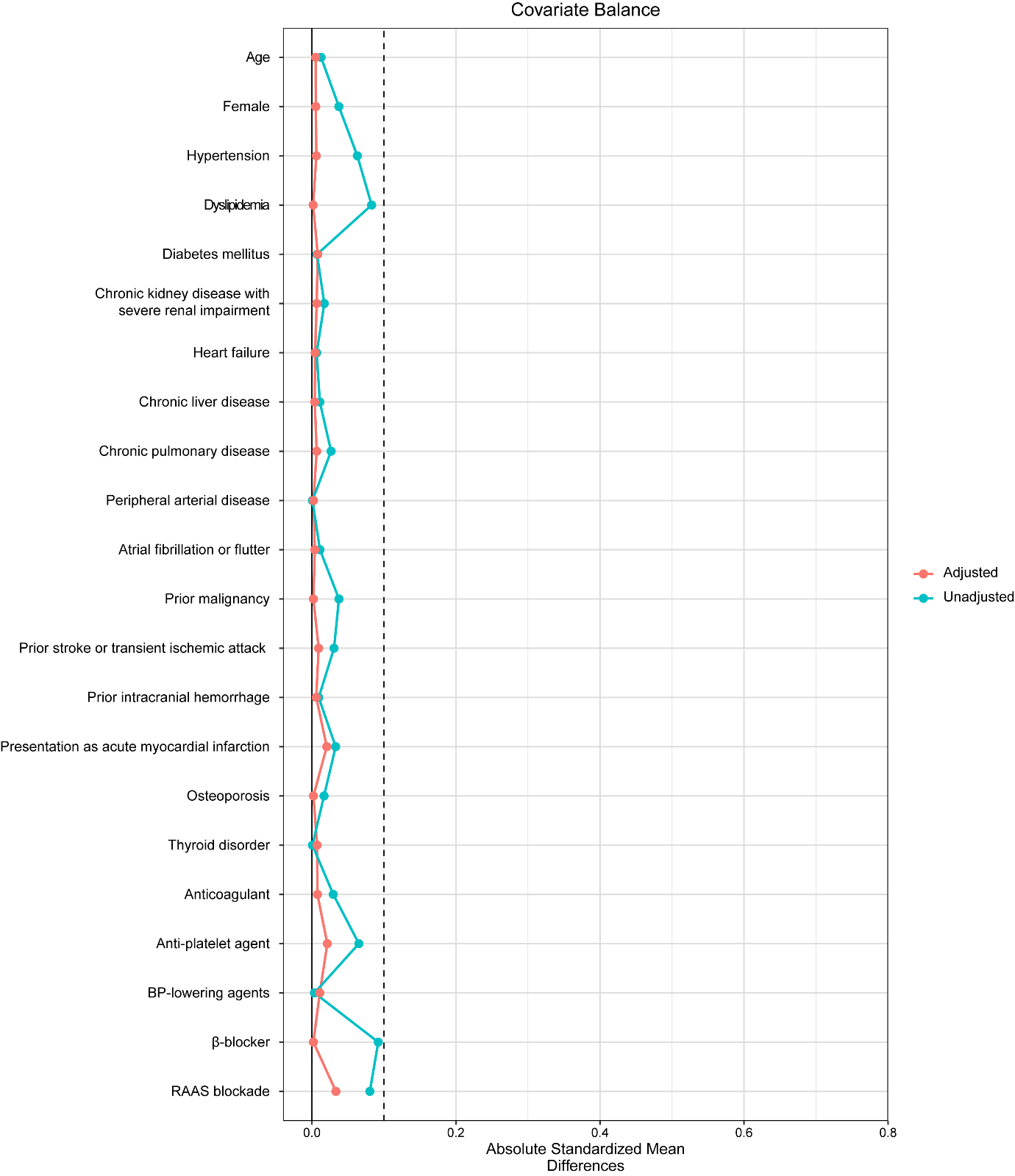
**

RAAS, renin–angiotensin–aldosterone system.

**Figure S2.** Distribution of stabilized inverse probability of treatment weighting. Density plot depicted as red color and green color indicates biodegradable polymer drug-eluting stents (BP-DES) and durable polymer drug-eluting stent (DP-DES), respectively.

**
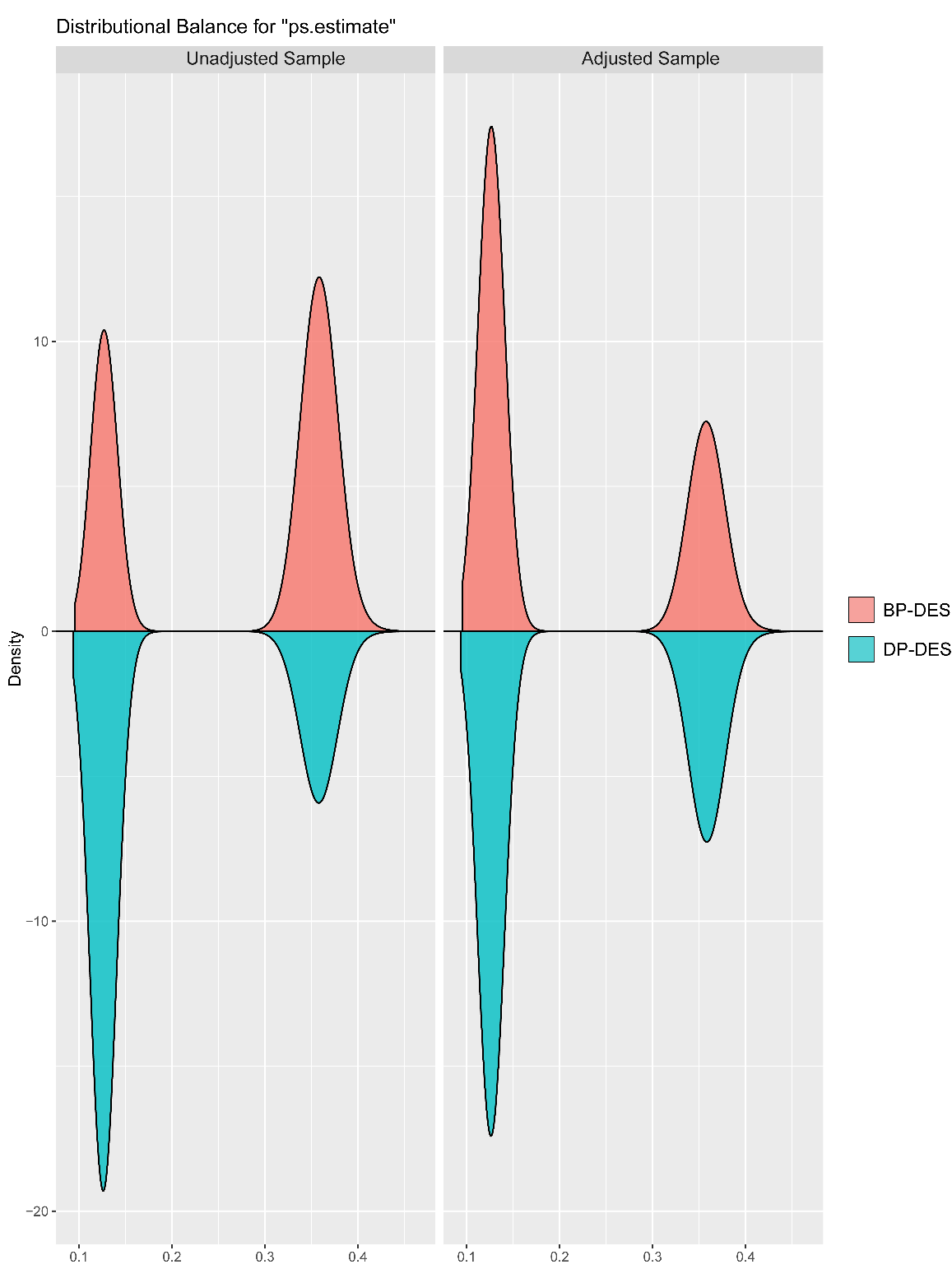
**

BP, biodegradable polymer; DP, durable polymer; DES, drug-eluting stent

**Figure S3. Subgroup analyses for myocardial infarction**

**
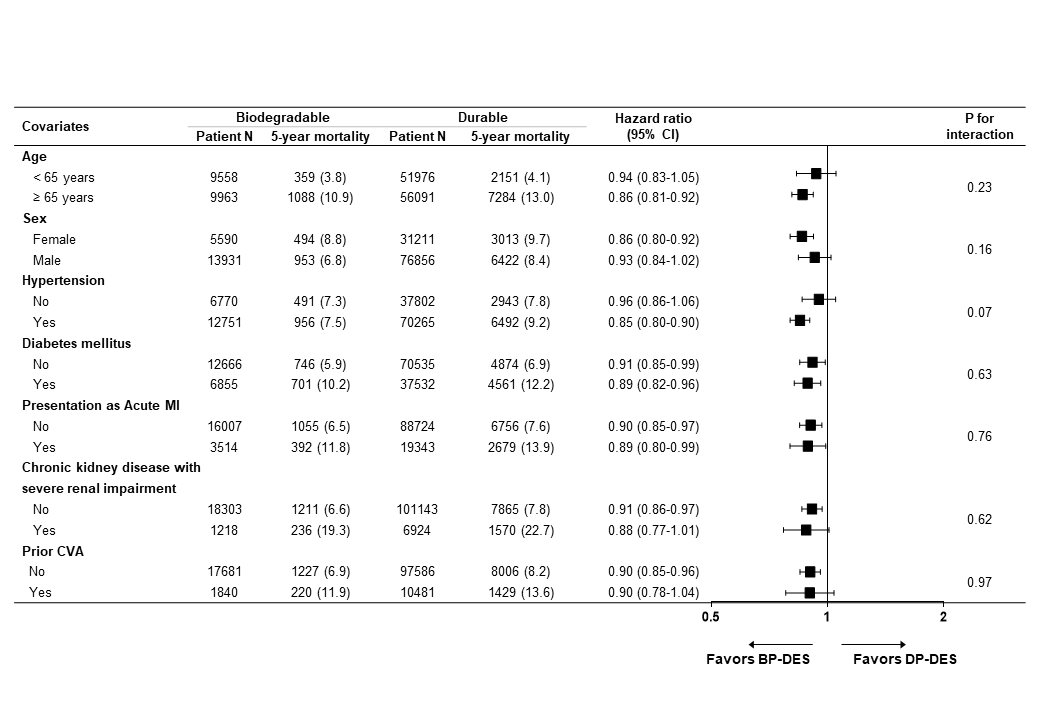
**

Numbers and percentages show the number of patients at risk, those who experienced myocardial infarction, and the relative risk of myocardial infarction at 5 years after drug-eluting stent implantation. CI, confidence interval; CVA, cerebrovascular accidents; BP-DES, biodegradable polymer drug-eluting stent; DP-DES, durable polymer drug-eluting stent; MI, myocardial infarction.
